# A novel mechanism underlying allosteric regulation of ADAMTS-13 revealed by hydrogen−deuterium exchange plus mass spectrometry

**DOI:** 10.1016/j.rpth.2022.100012

**Published:** 2022-12-13

**Authors:** Vikram G. Pillai, X. Long Zheng

**Affiliations:** 1 Department of Pathology and Laboratory Medicine, The University of Kansas Medical Center, Kansas City, USA; 2 Department of Biophysics, Institute of Medical Sciences, Banaras Hindu University, Varanasi, India; 3 Institute of Reproductive Medicine and Developmental Sciences, The University of Kansas Medical Center, Kansas City, USA

**Keywords:** ADAMTS-13 protein, allosteric regulation, deuterium exchange, conformation, ADAMTS-13 A disintegrin and metalloprotease with thrombospondin type 1 repeats-13, MDTCS A C-terminal truncated ADAMTS-13 variant after the spacer domain, T5C A C-terminal fragment of ADAMTS-13 consisting of thrombospondin type 1 repeat 5-8 and CUB domain., CUB The CUB domain for complement C1r/C1s, Uegf, Bmp1, DTCS An ADAMTS-13 variant consisting of a disintegrin, first TSP1 repeat, Cys-rich, and spacer domain., TTP Thrombotic thrombocytopenic purpura, HX-MS hydrogen–deuterium exchange plus mass spectrometry, ULVWF Ultra large von Willebrand factor

## Abstract

**Background:**

ADAMTS-13, a plasma metalloprotease, cleaves von Willebrand factor. ADAMTS-13 activity appears to be regulated through allosteric inhibition by its distal C-terminus.

**Objectives:**

The objective of this study was to better understand how domain–domain interactions may affect ADAMTS-13 conformations and functions.

**Methods:**

We performed deuterium–hydrogen exchange plus mass spectrometry to assess the number and rate of deuterium incorporation into various peptides of full-length ADAMTS-13 and its truncated variants.

**Results:**

Under physiological conditions, a bimodal distribution of deuterium incorporation was detected in the peptides from metalloprotease (217-230 and 282-304), cysteine-rich (446-482), and CUB (for complement C1r/C1s, Uegf, Bmp1) domains (1185-1214, 1313-1330, 1341-1347, 1358-1378, and 1393-1407) of full-length recombinant ADAMTS-13, but not of truncated variants. These results suggest that the full-length ADAMTS-13 undergoes conformational changes. On removal of the middle and distal C-terminal domains, the number and rate of deuterium incorporation were increased in the peptides from cysteine-rich (445-467, 467-482, and 495-503) and spacer domains (621-642 and 655-654) but decreased in the peptides from metalloprotease (115-124, 217-230, and 274-281). Moreover, most peptides, except for 217-230 and 1357-1376, exhibited a pD-dependent deuterium incorporation in the full-length ADAMTS-13, but not in the truncated variant (eg, MDTCS or T5C). These results further suggest that the bimodal deuterium incorporation observed in the peptides from the full-length ADAMTS-13 is the result of potential impact from the middle to distal C-terminal domains. Surface plasmon resonance revealed the direct binding interactions between the distal and proximal domains of ADAMTS-13.

**Conclusion:**

Our results provide novel insight on how intramolecular interactions may affect conformations of ADAMTS-13, thus regulating its proteolytic functions.

## Essentials

• The mechanism underlying allosteric regulation of ADAMTS-13 activity is not fully understood.
• Biophysical study reveals conformational changes of ADAMTS-13 after truncation or pH changes.
• Biophysical study also detects direct binding between distal and proximal ADAMTS-13 domains.
• Our findings provide insights into how interdomain interactions affect ADAMTS-13 conformation.

## Graphical abstract

**Figure:**
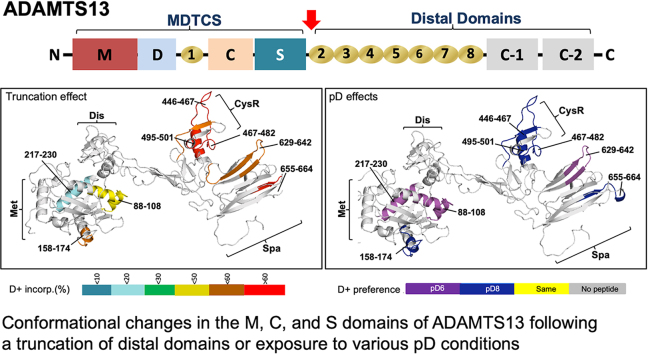

## 1 Introduction

A multidomain and zinc-dependent metalloprotease ADAMTS-13 consists of proximal and distal segments. The proximal segment includes a metalloprotease, a disintegrin-like domain, the first thrombospondin type 1 repeat, a cysteine-rich, and a spacer domain. The distal segment contains 7 more TSP1 repeats and 2 CUB domains (for complement C1r/C1s, Uegf, Bmp1) [1,2]. ADAMTS-13 is synthesized in hepatic stellate cells [3,4], endothelial cells [[4], [5], [6]], and perhaps megakaryocytes [7] and is released into the blood stream, where it cleaves newly released ultra large von Willebrand factor (ULVWF) [8,9]. The cleavage of ULVWF by ADAMTS-13 occurs specifically at the Tyr^1605^-Met^1606^ bond [10]. This proteolytic cleavage is essential for the regulation of von Willebrand factor (VWF) adhesive function and thrombus formation at the sites of vascular injury.

Severe deficiency of plasma ADAMTS-13 activity, primarily resulting from acquired autoantibodies against ADAMTS-13, may lead to a potentially fatal syndrome thrombotic thrombocytopenic purpura (TTP) [11,12]. Mild to moderate deficiency of plasma ADAMTS-13 activity is associated with many inflammatory and thrombotic disorders, including myocardial infarction [13,14], ischemic stroke [15,16], malignant malaria [[17], [18], [19]], preeclampsia [20,21], and acute renal insufficiency in critically ill patients [22] and severe COVID-19–associated thrombosis [[23], [24], [25]].

ADAMTS-13 activity is primarily regulated at its substrate level through fluidic shear [10,26], which causes stretch and conformational changes in the central A2 domain of VWF, allowing ADAMTS-13 to access to the cleavage site. Our previous studies have demonstrated that binding of protein cofactors such as coagulation factor VIII [27], platelet glycoprotein 1b [28,29], and apoB100/LDL [30] may facilitate shear-induced conformational changes that accelerate the cleavage of VWF by ADAMTS-13. More recently, several studies have also demonstrated that ADAMTS-13 in solution may exhibit various conformations [31,32], and at least, some are sensitive to the changes in environmental pH [32], metal ions (Ca^2+^ and Zn^2+^) [33,34], and the interaction with VWF substrate [32,35] or antibodies in patients with TTP [32,36]. Additionally, a removal of its distal segment consisting of TSP-1 2 to 8 repeats and CUB domains may also result in dramatic conformational changes, thus increasing ADAMTS-13 activity toward the VWF substrates [32]. Moreover, point mutations in the spacer domain may also activate ADAMTS-13 by disrupting the interaction between the proximal and distal domains [36,37]. However, the data available thus far are largely functional and indirect. The exact mechanism underlying how the domain–domain interactions may affect overall conformation of ADAMTS-13 protein is not known.

We therefore used the cutting-edge hydrogen–deuterium exchange plus mass spectrometry (HX-MS) to determine dynamic changes in protein conformation of various peptides derived from a full-length ADAMTS-13 protein and two truncated ADAMTS-13 variants (the N-terminal fragment and the C-terminal fragment) under various conditions. Our results provide direct and high-resolution evidence indicating how the domain–domain interactions may affect the overall conformations and functions of ADAMTS-13 protease.

## 2 Methods

### 2.1 Recombinant full-length ADAMTS-13 and its truncated variants

A full-length recombinant ADAMTS-13 (FL-A13) was expressed in human embryonic kidney (HEK)-293 cells (Invitrogen, Carlsbad, CA) [38]. N-terminal ADAMTS-13 fragment MDTCS (aa1-685) and C-terminal ADAMTS-13 fragment T5C (aa884-1427) were expressed in Drosophila (S2) cell line (Invitrogen) [39] and Chinese hamster ovary cell line [40] (Invitrogen), respectively. The construct consisting of a disitegrin, the first thrombospondin type 1 repeat, a cysteine-rich and a spacer domain (ie, DTCS) and CUB for complement C1r/C1s, Uegf, and Bmp1 were expressed in HEK-293 cells [41]. All purified proteins were analyzed by 5% to 20% SDS-acrylamide gel electrophoresis with Coomassie blue staining.

### 2.2 HX-MS basic

**Figure 1:**
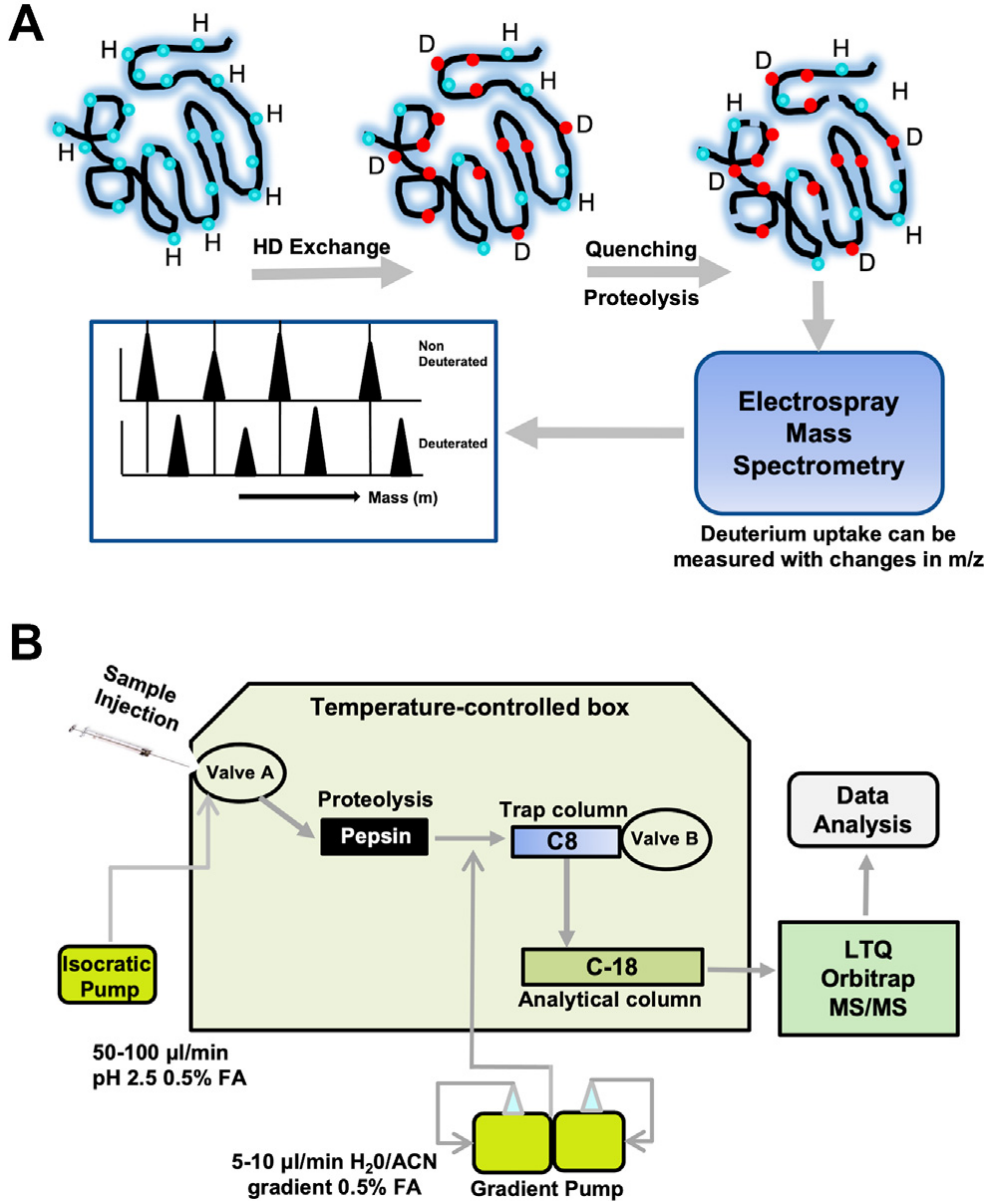
The principle and procedure of hydrogen–deuterium exchange plus mass spectrometry. (A) Schematic diagram illustrating the principal of hydrogen–deuterium exchange (HX) plus mass spectrometry (MS) for the detection of deuterated and non-deuterated peptides following protease digestion. (B) Schematic presentation of the setting and workflow of HX-MS. An isocratic pump maintains a constant pressure and allows the quenched sample to pass through the proteolytic (eg, pepsin) column. Then, a gradient pump pushes digested peptides into a C8-trap column, which collects all peptides and separates through a C-18 analytic column before being sprayed onto the detector of LTQ Orbitrap XL mass spectrometer. All procedures are performed in the temperature-controlled box (0-4ºC).

The HX-MS is a technique used to elucidate protein-protein interaction, dynamics, and conformational changes [[42], [43], [44]]. It measures the isotopic mass change associated with the protein amide backbones with its surrounding, which depends on the overall folding and dynamics of the protein. During labeling, proteins are exposed to deuterated buffer for various times to allow deuterium incorporation into their backbone. An aliquot is then quenched by lowering the pH to 2.5 and temperature to nearly zero, followed by a protease digestion. Desalted proteolytic peptides with the help of chilled reverse phase high performance liquid chromatography are then eluted and sprayed onto a mass spectrometer. Analysis of the MS/MS data reveals the difference in mass due to deuterium uptake. Other than structural rearrangement, the H–D exchange rate also depends on temperature, pH, and nature of the amino acid. In addition, it depends on the nature of secondary structure, H-bonding, and solvent accessibility. The H–D exchange rate is relatively lower in the obstructed core areas of the protein than that in the exposed areas (Figure 1A).

### 2.3 HX-MS experiments and data analyses

Purified recombinant FL-A13, MDTCS, and T5C were diluted in all H-buffer at pH7.0 and incubated with a buffer containing D_2_O for D-labeling for 0 to 6 hours. In addition, proteins were diluted into a 100% D_2_O buffer at pD7.0 containing 10 mM sodium phosphate or a 100% H_2_O buffer at pH7.0 containing 10 mM potassium phosphate and 150 mM sodium chloride at a final volume of 32 μL. At different exchange time points (10, 60, 600, and 6000 seconds), an ice-cold quenching buffer (1.2 M GdHCl, 54 mM TCEP, 30 mM glycine, pH2.5) was added to stop the reaction. All D labeling was achieved by mixing the sample at 60 ºC for 30 minutes. The quenched sample was then passed through a protease column containing either a pepsin or fungal protease, under an isocratic flow, to a small C-8 trap column (1 × 5 mm, 5-μm beads), then gradient eluted [9 μL/min, 10–50% (experiment one) or 40% (experiment 2) (vol/vol) acetonitrile over 14 m] to a C-18 analytical column (0.3 × 50 mm, 3-μm beads). The digested peptides were than eluted from the C-18 column and electro sprayed into an LTQ Orbitrap XL mass spectrometer for a second dimension of separation. For different pD experiments, a full-length and a truncated variant was diluted into a 100% D_2_O buffer at 2 different pDs (6.0 and 8.0) with 10 mM sodium phosphate in a final volume of 32 μL. After an indicated incubation time (60 seconds for pD8.0 vs. 6000 seconds for pD6.0), an ice-cold quench buffer (5.4 M GdHCl, 240 mM TCEP, 134 mM acidified glycine, pH2.5) was added. The protein was allowed to pass through the protease column and was later electro sprayed onto a mass spectrometer as described above (Figure 1B). Peptides were identified and analyzed for carrying D-label by the ExMS program at peptide resolution [[43], [44], [45], [46]].

### 2.4 Surface plasmon resonance (SPR)

A purified CUB domain fragment was immobilized on a CM5 sensor chip (GE Healthcare) through amine-coupling. A N-terminal ADAMTS-13 fragment (ie, MDTCS or DTCS or MDT) at various concentrations were perfused over the CUB-immobilized surface at 30 μl/min for 120 seconds. Protein dissociation was followed for 300 seconds. The surface was regenerated with 10 mM of glycine at pH2.0 for 30 seconds, and followed by perfusion with a re-equilibration buffer for another 30 seconds prior to next round of binding assay. An equilibrium dissociation constant (*K*_*D*_) was determined by Prism 8.0 software (GraphPad) using the maximal response unit (RU) as a function of increasing concentrations of the ligands [47].

## 3 Results

### 3.1 Constructs and characterization of recombinant full-length ADAMTS-13 and its truncated variants

**Figure 2:**
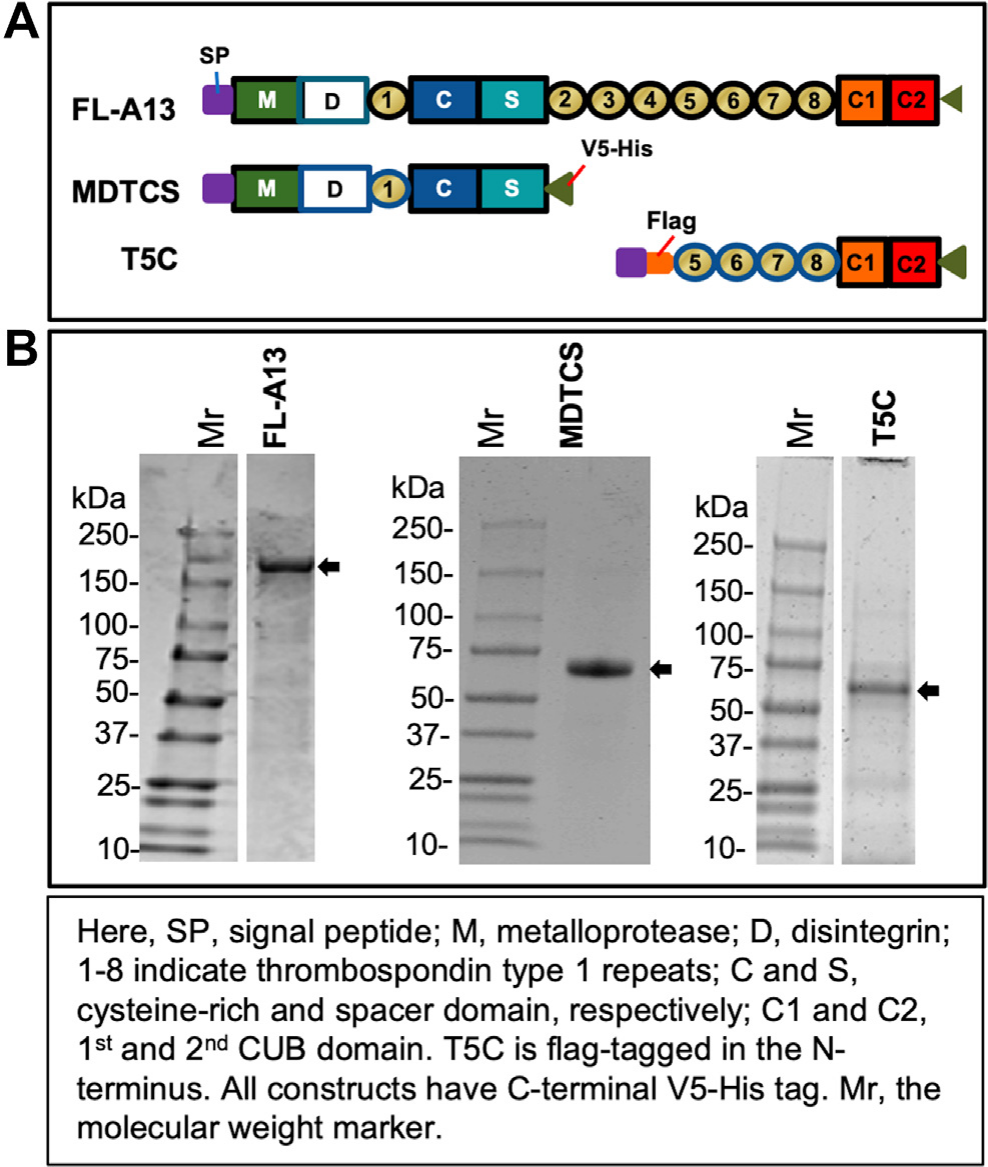
Constructs and purified full-length ADAMTS13 and variant proteins. (A). Schematic representation of the domain structures of a full-length ADAMTS13 and two truncated variants. (B). SDS-polyacrylamide gel with Coomassie blue staining demonstrates the purified recombinant full-length ADAMTS13, MDTCS, and T5C, ∼5 μg per lane (arrowhead).

Figure 2A shows the schematic representation of the constructs used to express FL-A13 and its truncated variants (ie, MDTCS and T5C) for the HX-MS experiments. All recombinant proteins were expressed in HEK293 or S2 cells and purified to homogeneity according to the methods described elsewhere [41,[47], [48], [49]]. Purified FL-A13 and MDTCS, but not T5C, exhibited proteolytic activity toward FRETS-VWF73 and multimeric VWF as expected (not shown). Figure 2B shows the purified proteins revealed by the sodium dodecyl sulfate–polyacrylamide gel (SDS-PAGE) with Coomassie blue staining. The band with molecular weights of ∼195 kDa, ∼72 kDa, and ∼55 kDa correspond to FL-A13, MDTCS, and T5C, respectively [50]. Other ADAMTS-13 fragments used in the binding experiments (eg, MDT, DTCS, and CUB) were described in our previous publications (not shown) [40,41,47].

### 3.2 Peptide coverage of the full-length ADAMTS-13 and its truncated variants by mass spectrometry

**Figure 3:**
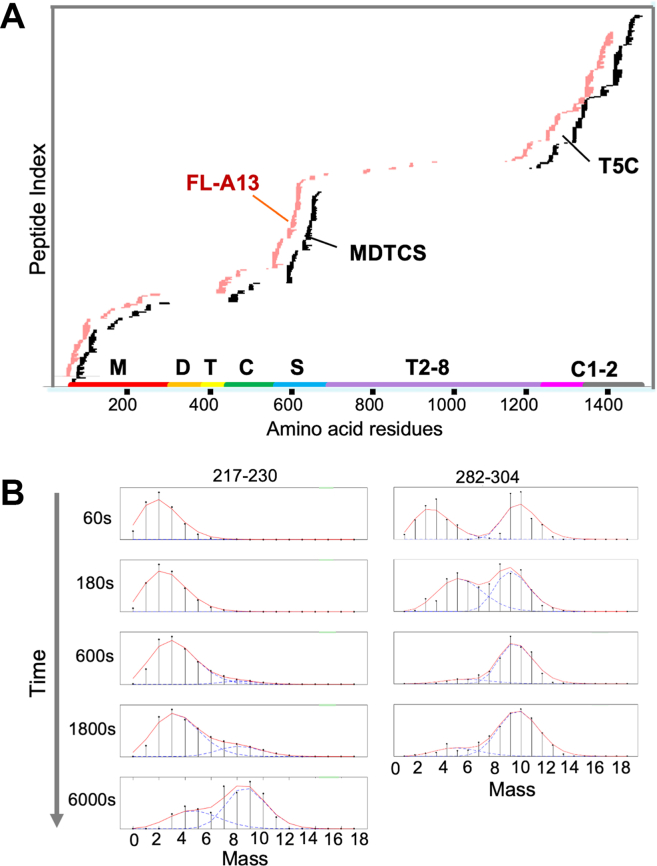
Peptide coverage and representative illustration of single and bimodal deuterium incorporations. (A). Overall peptide coverage in all H of a full-length ADAMTS13 (FL-A13) (pink) and a truncated ADATMS13 variant (eg, MDTCS or T5C) (black) following protease digestion, column chromatography, and mass spectrometric analysis. (B). Representative illustration of bimodal deuterium incorporations in peptides (eg, 217-230 and 282-304) of a full-length ADAMTS13 at pD7.0. From top to bottom is the exchange occurring from 10 to 6000 seconds.

To assess the coverage of peptides, protease digestion, liquid chromatography, and MS, as illustrated in Figure 3A based on the design from Dr. Englander’s laboratory [42,51], were performed. The preliminary experiments were conducted under all H-conditions at pH7.0. As shown, recombinant full-length ADAMTS-13 protein yielded ∼460 unique and/or overlapping peptides that covered the metalloprotease domain (M), the disintegrin-like domain (D), the CysR (C), and spacer domains (S) (Figure 3B). This proximal segment of ADAMTS-13 is essential for recognition and proteolytic cleavage of VWF [40,41,[52], [53], [54]] and is predominantly targeted by autoantibodies that cause immune-mediated TTP [[55], [56], [57]]. Only 77 and 23 unique and/or overlapping peptides were recovered from the truncated ADAMTS-13 variants MDTCS and T5C, respectively (Figure 3A). Unfortunately, fewer peptides were recovered from the disintegrin domain, the CysR domain, and the first TSP1 repeat (Figure 3B), likely resulting from glycosylation and/or lack of appropriate protease cleavage site, which may prevent from accurate peptide identification by MS and software.

### 3.3 Time-dependent incorporation of deuterium into peptides from a full-length ADAMTS-13 and its truncated variants at neutral pD

To assess the dynamic incorporation of deuterium into the peptides of a full-length ADAMTS-13 vs. its truncated variants, purified recombinant FL-A13, MDTCS, and T5C were incubated with 10 mM sodium phosphate buffer containing deuterium (D_2_O) for 10, 60, 600, and 6000 seconds at pD7.0. The reaction was terminated by lowering the pD and chilling at 0 ºC. Deuterium and hydrogen exchange rate is the lowest around pH/pD2.5 and low temperature. Addition of quench buffer stops the deuterium labeling and minimizes the back exchange. Moreover, at pH >2.5, the rate of hydrogen and deuterium exchange increased logarithmically as the pH/pD increases. Because of this, the rate of exchange at pH6.0 is 10× slower than that at pH7.0 and 100× slower than that at pH8.0. Following the protease digestion, peptides were separated with liquid chromatography and identified by mass spectrometer. As shown, a time-dependent bimodal change of mass spectra was detected in various peptides of the metalloprotease domain (217-230 and 282-304) (Figure 3B), the CysR domain (446-482 and 446-502), and the CUB domains (1185-1214, 1313-1330, 1341-1346, 1358-1378, and 1393-1407) with multiple overlapping peptides (not shown). These bimodal deuterium incorporation patterns were only observed in the peptides derived from the full-length ADAMTS-13 protein, but not from the truncated variant (ie, MDTCS or T5C) (Table 1). The results suggest that domain–domain interactions between the proximal and distal segments may affect the overall conformations of ADAMTS-13 and thus its function.

**Table 1:** Peptides exhibiting a bimodal deuterium incorporation pattern in a full-length ADAMTS-13, but not in MDTCS and T5C fragments at neutral pD conditions.

Domain	Peptide	Amino acid residues
Met	217-230	DLGVTIAHEIGHSFG,
	282-304	WDPPRPQPGSAGHPPDAQPGLYY
CysR	446-482	MSQQCARTDGQPLRSSPGGASFYHWGAAVPHSQGDALC
CUB1	1185-1214	SVQSSACGRQHLEPTGTIDMRGPGQADCAV
CUB2	1313-1330	SLSPATSNAGGCRLFINV
	1341-1347	LATNMGAGT
	1358-1378	IRDTHSLRTTAFHGQQVLYW
	1393-1407	LKAQASLRGQYWTLQS

CUB1, the 1st CUB domain; CUB2, the second CUB domain; CysR, cysteine-rich domain; MDTCS, an ADAMTS-13 variant truncated after spacer domain; Met, metalloprotease domain; T5C, C-terminal fragment consisting of TSP1 5-8 and two CUB domains.

### 3.4 The C-terminal truncation results in altered conformation of the N-terminal domains

**Figure 4:**
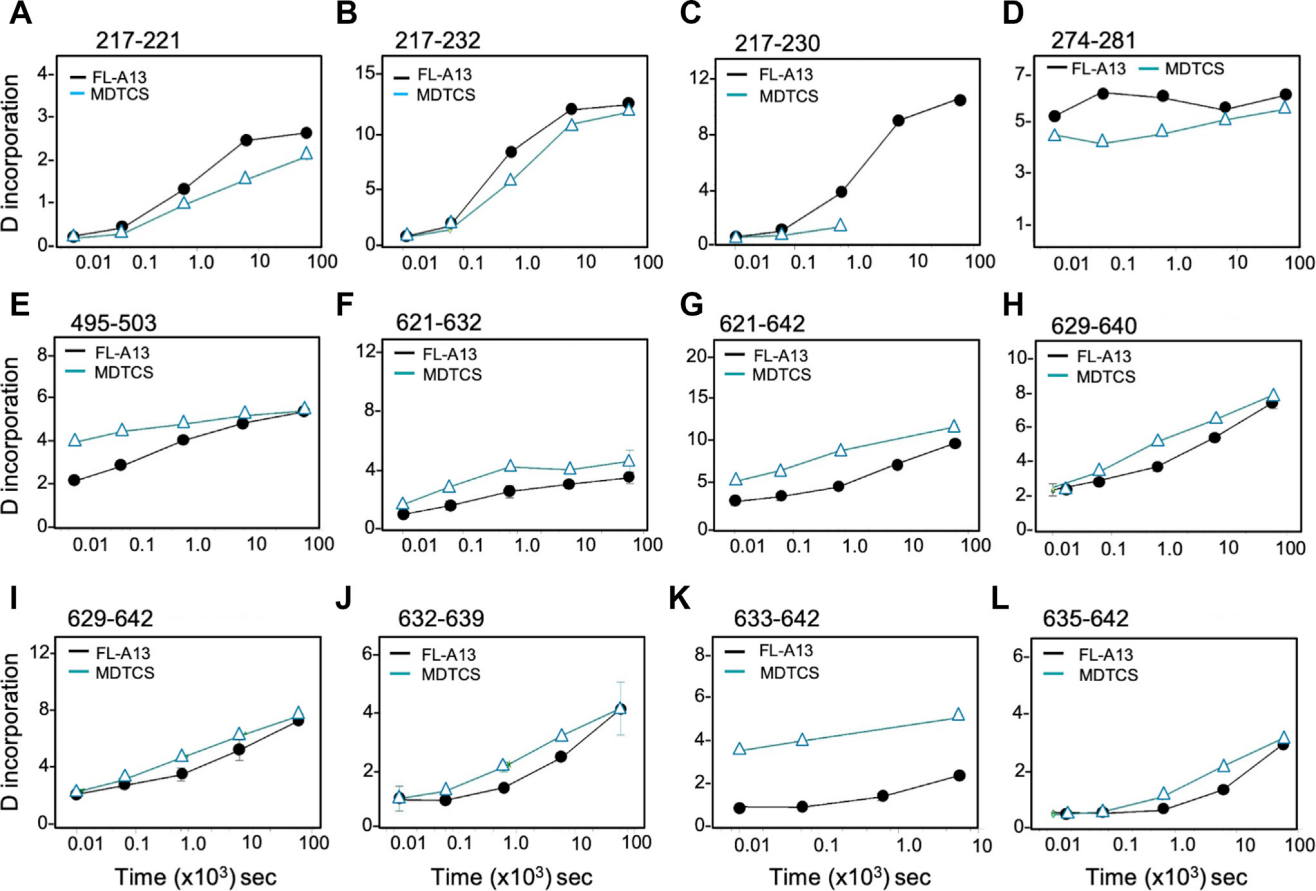
Time-dependent incorporation of deuterium in various peptides from a full-length ADAMTS13 and a MDTCS fragment. Each panel demonstrates the rate of deuterium incorporation as a function of time (10-6000 seconds) in various peptides (as labeled in a-l) derived from a full-length ADAMTS13 (ie, FL-A13) (closed circle and black line) and a truncated ADAMTS13 variant (ie, MDTCS) (open triangle and cyan line). Images a to d show the peptides derived from the metalloprotease domain; e, from the Cys-rich domain; and f to l, from the spacer domain.

In addition to the difference in the bimodal deuterium incorporation into the peptides in the full-length and the truncated ADAMTS-13 variant (MDTCS), a significant increase in the initial deuterium incorporation or the rate of deuterium incorporation as a function of time in the peptides, exemplified in Figure 4, was observed in the peptides derived from the CysR domain (eg, 445-467, 467-482, and 495-503) and the spacer domain (eg, 621-642 and 633-642 or 655-654), domains of the truncated MDTCS variant compared with those derived from the full-length ADAMTS-13 protein (Table 2 and Figure 4).

**Table 2:** Deuterium uptake in the peptides from MDTCS or T5C in comparison to those from a full-length ADAMTS-13 at pD7.0.

Domain	Peptides	Relative deuterium uptake
**Met**	88-108	Same
	158-165	Decreased by 30%
	217-230	Decreased by 50%
**CysR**	445-467	Increased by 40%
	467-482	Increased by 10-20%
**Spa**	494-503	Increased by 10%
	621-642	Increased by 20%
	655-664	Increased by 20%
**CUB1**	1214-1225	Increased by 10%
	1261-1268	Increased by 20%
	1283-1293	Increased by 20%
**CUB2**	1303-1311	Increased by 30%
	1359-1376	Increased by 20%
	1377-1387	Increased by 10%
	1395-1400	Same

CUB1 and CUB2 are the first and second CUB domains, respectively.

MDTCS, ADAMTS-13 variant truncated after spacer domain; Met, metalloprotease domain; CysR, Cysteine-rich domain; Spa, spacer domain; T5C, C-terminal fragment containing TSP1 5-8 and CUB domains.

### 3.5 Identification of amino acid residues in the spacer domain that are differentially exchanged in the full-length vs. truncated ADAMTS-13 variant

**Figure 5:**
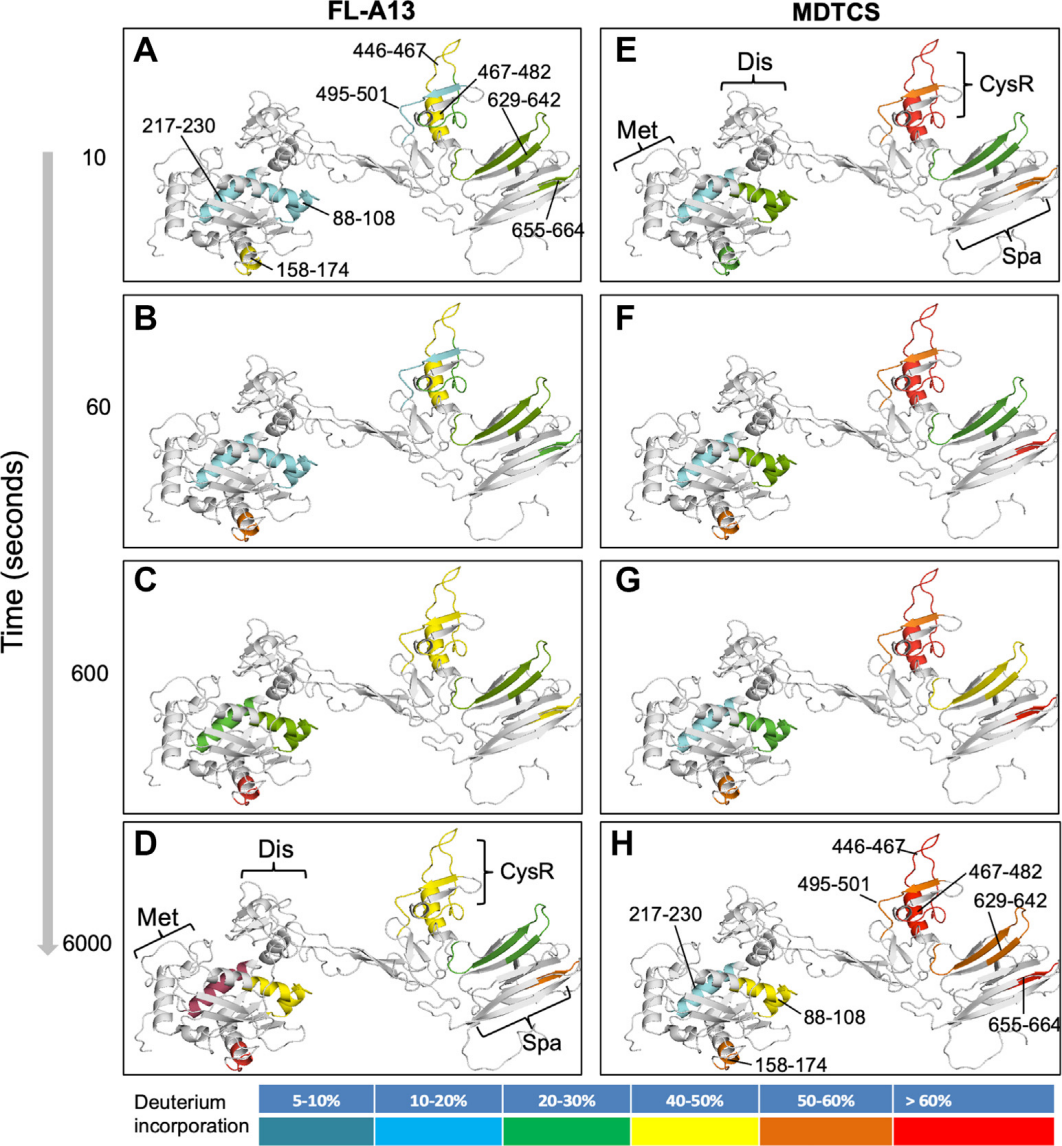
Ribbon representation of the peptides with altered deuterium incorporation rates in the M-D-T-C-S domains of a full-length ADAMTS13 comparing with the same regions in a truncated MDTCS variant. The M-D-T-C-S domains (as indicated in the bottom on the left column and on the top in the right column) demonstrate the peptides involving in the differential deuterium incorporation rates as a function of time (10 to 6000 seconds) from top to bottom in a full-length ADAMTS13 (left) and a truncated MDTCS fragment (right). The color from blue to red indicates that the percentage of update of deuterium increases from 5% to 10% to >60%. Here Met, Dis, CysR, and Spa indicate metalloprotease, disintegrin, cysteine-rich, and spacer domains, respectively.

**Figure 6:**
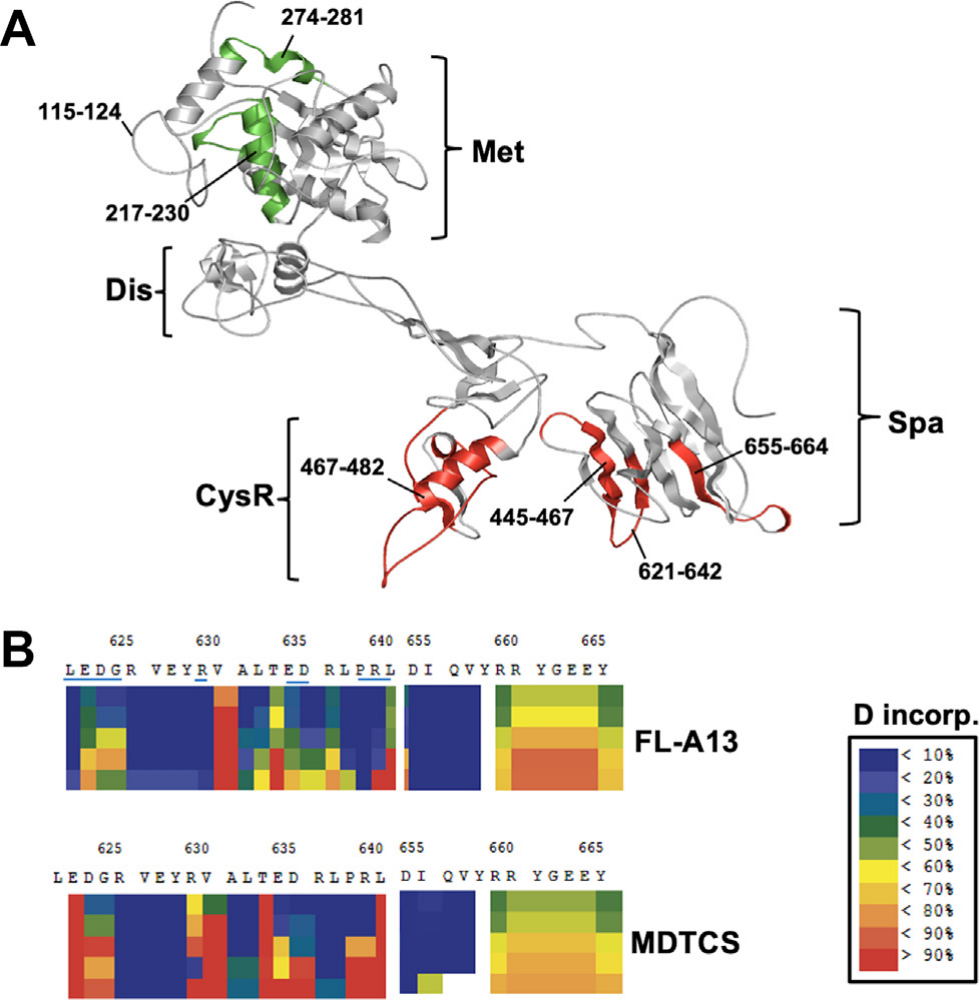
Detailed analysis of deuterium incorporation at a single-residue resolution in the spacer domain. (A) A ribbon representation of low and high rates of deuterium incorporation in the peptides derived from the metalloprotease (Met), Cysteine-rich (Cys-R), and spacer domains (Spa) in the truncated MDTCS compared with that in the peptides in the full-length ADAMTS13 at a neutral pD. (B) A heat map demonstrates differential rates of deuterium incorporation in the portion of spacer domain of the full-length ADAMTS13 (top) and MDTCS fragment at pD7.0. The percentage of uptake ranges from blue (<10% D uptake) to red (>90% D uptake). No back-exchange correction and statistical filtering were performed for the generation of heat map.

Further analysis using the overlapping peptides allowed us to identify the specific amino acid residues (highlighted in red or underlined) in the CysR and/or spacer domains of the MDTCS fragment with an increased or accelerated rate (eg, LEDG, R, ED, and PR) of deuterium incorporation compared with those in the full-length ADAMTS-13 protein at neutral pD (Figures 5 and 6).

Together, these results demonstrate dramatic alterations of deuterium accessibility, indicative of conformational changes in various discrete regions of ADAMTS-13, after its being split into 2 fragments. The findings suggest that direct domain–domain interactions may occur between the proximal and the distal segments of ADAMTS-13, which contributes to the overall stable ternary structure under physiological conditions.

### 3.6 Conformational changes in ADAMTS-13 resulting from alteration of pD

**Figure 7:**
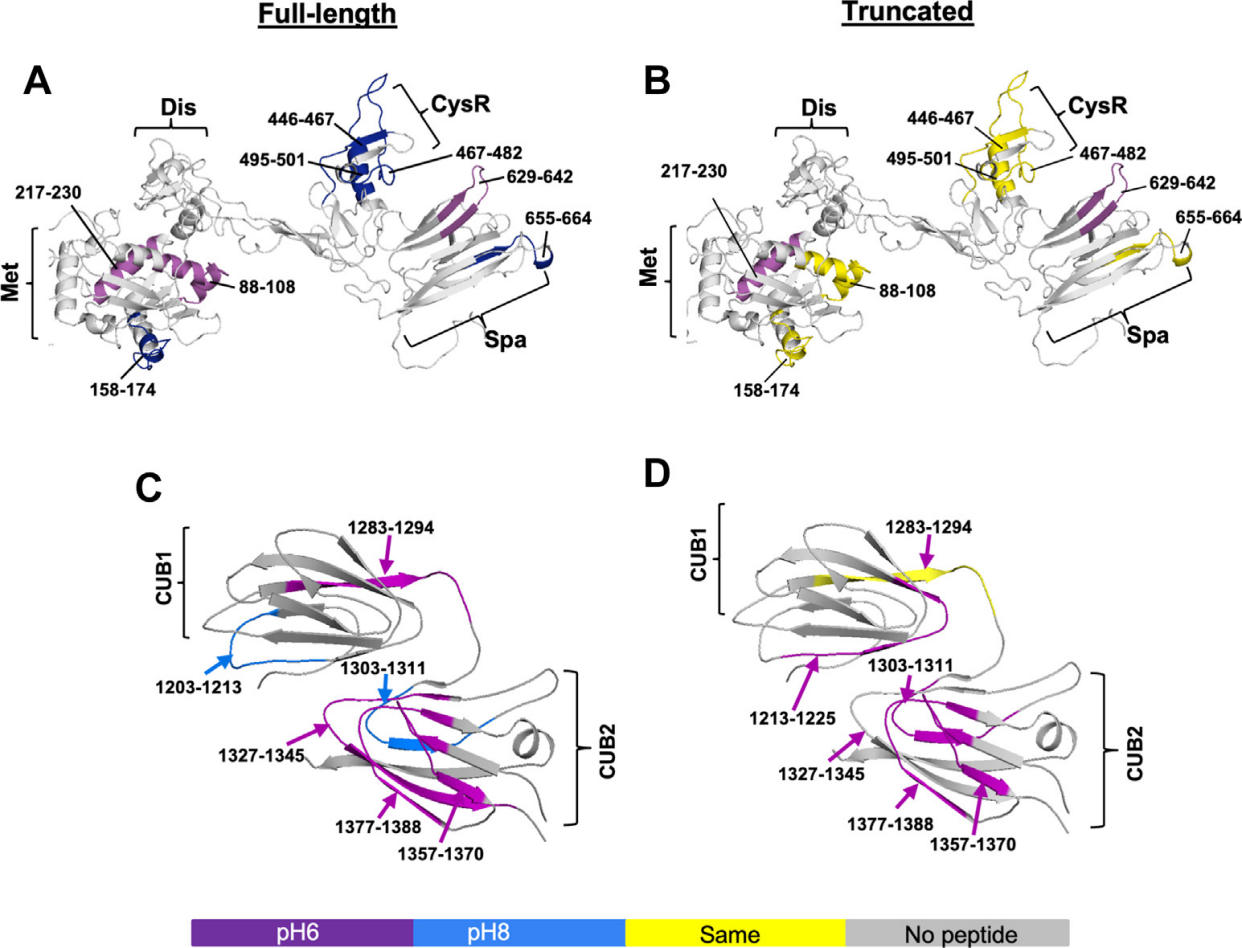
Deuterium incorporation in the peptides from the full-length ADAMTS13 and the truncated variants at pD6.0 over time in comparison to that of the same peptides at pD8.0. The deuterium incorporation rates in the peptides derived from the N-terminal portion the full-length ADAMTS13 (A) and the MDTCS fragment (B) at pD6.0 compared with that at pD8.0 at 2 different time points. In addition, the deuterium incorporation rates of the peptides derived from C-terminus of a full-length ADAMTS13 (C) and T5C (D) at pD6.0 compared with that of the same peptides at pD8.0 at 2 different time points. The deuterium incorporation rate is approximately 100× slower at pD6.0 than at pD8.0. Thus, the comparison of the deuterium incorporation was made between 6000 seconds at pD6.0 and 60 seconds at pD8.0, for instance. Here, Met, Dis, CysR, Spa, CUB1, and CUB2 indicate metalloprotease, disintegrin, cysteine-rich, spacer, CUB-1 and CUB-2 domains, respectively. The purple or blue colors indicate an increase of deuterium incorporation rate at pD6.0 compared with pD8.0. Yellow indicates the peptides with same deuterium incorporation rate at pD6.0 and pD8.0. Gray shows the areas of no peptides recovered.

Changing pH from 8.0 to 6.0 results in the activation of plasma ADAMTS-13, which increases its proteolytic activity by approximately 3-fold [32]. This degree of ADAMTS-13 activity increase is similar to that resulted from the C-terminal truncation [32]. To determine whether pD change affects ADAMTS-13 conformations, we performed the direct comparison of deuterium uptake in various peptides derived from a full-length ADAMTS-13 and its truncated variants at pD8.0 and pD6.0 at the comparable incorporation time scale. As shown, the deuterium incorporation rate increases or decrease in the peptides in the metalloprotease, CysR, and spacer domains as well as in the CUB domains of full-length ADAMTS-13 as the pD value drops from 8.0 to 6.0 (Table 3 and Figure 7), consistent with pD-induced conformational changes in these areas; however, when the deuterium incorporation experiments were performed with the MDTCS or T5C fragment, most, if not all, pD-associated deuterium incorporation rate changes disappeared, suggesting no pD-induced conformational changes in these peptides derived from the N- and C-terminal fragments. These results further support the role of distal C-terminal domains in modulating the conformation of the proximal N-terminal domains of ADAMTS-13.

**Table 3:** Deuterium incorporation rates over time in peptides from a full-length ADAMTS-13 and its truncated variant (MDTCS or T5C) at pD6.0 in comparison to that at pD8.0.

Domain	FL-A13		MDTCS or T5C	
	Peptides	D-incorporation at pD6.0	Peptides	D-incorporation at pD6.0
**Met**	88-108	Increase	88-108	No change
	158-74	Decrease	166-174	No change
	217-230	Increase	217-230	Increase
**CysR**	447-482	Decrease	446-467	No change
	467-482	Decrease	468-482	No change
**Spa**	495-501	Decrease	494-502	No change
	629-642	Increase	621-642	No change
	655-664	Decrease	658-664	No change
**CUB-1**	1202-1213	Decrease	1213-1225	Increase
	1283-1294	Increase	1283-1294	No change
**CUB-2**	1303-1311	Decrease	1303-1311	Increase
	1357-1376	Increase	1359-1370	Increase

CUB-1 and CUB-2 are the first and second CUB domains, respectively.

CysR, cysteine-rich domain; FL-A13, full-length ADAMTS-13; MDTCS, a variant truncated after spacer domain; Met, metalloprotease; Spa, spacer domain; T5C, C-terminal fragment containing TSP1 5-8 repeats and CUB domains.

### 3.7 Direct binding interactions between the proximal and distal segments of ADAMTS-13 by surface plasmon resonance

**Figure 8:**
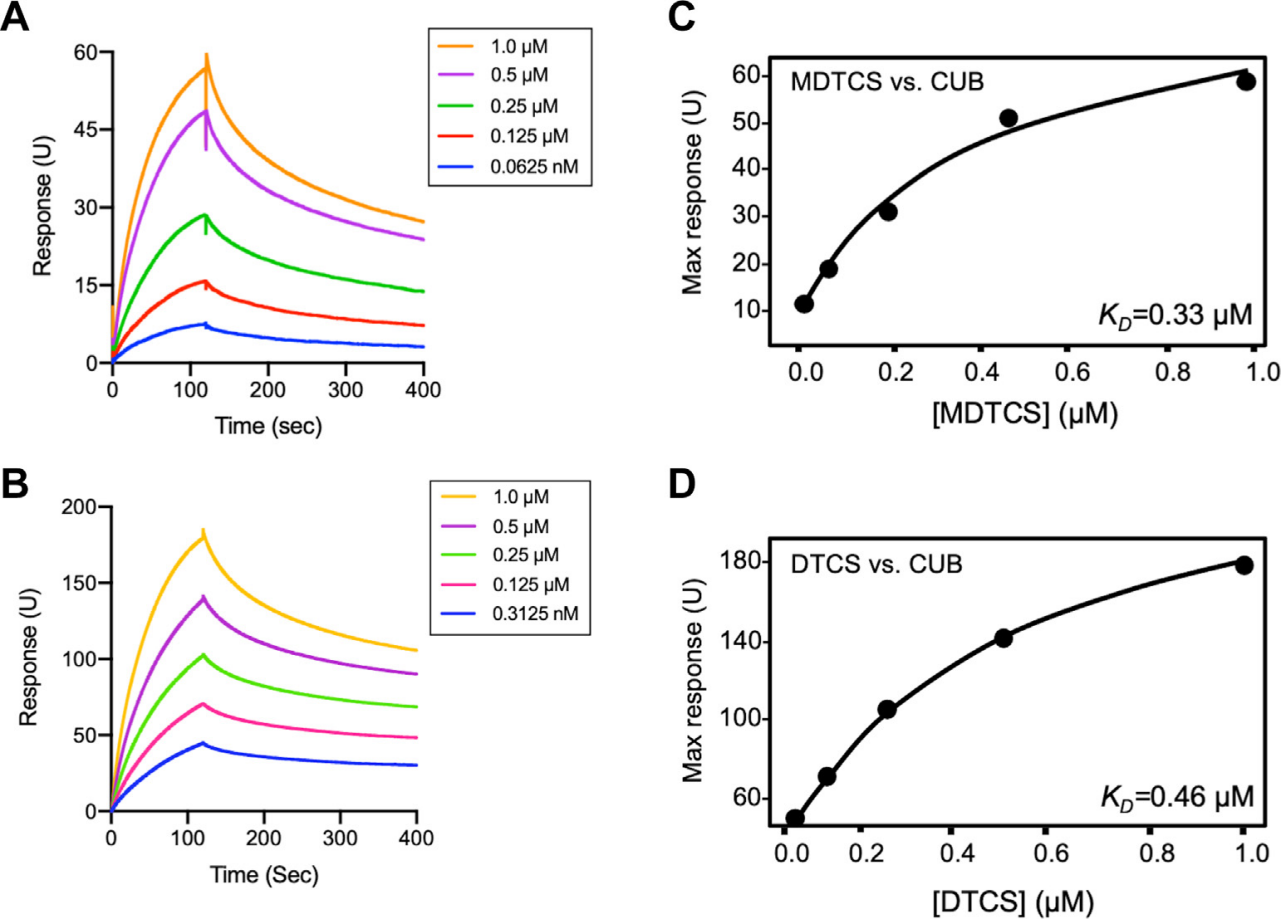
Binding interactions between N-terminus and C-terminus of ADAMTS13. (A) and (B) show the representative traces of the association and dissociation rates as a function of time following an injection of a purified recombinant MDTCS and DTCS fragment, respectively, over a CM5 surface that was immobilized with a purified CUB fragment. (C) and (D) are the means of maximal response units (RU) as a function of the concentration (0-1.0 μM) of the recombinant MDTCS (*K*_*D*_=0.33 μm and Chi^2^, 5.84) and DTCS (*K*_*D*_=0.46 μm, Chi^2^ 4.34), respectively. *K*_*D*_, equilibrium dissociation constant, was determined using the Prism 8.0 software.

Small angle X-ray, cryo-EM, and recent molecular docking data suggest that the distal domains may somehow fold back to interact with the proximal domains [32,58,59]. However, the direct evidence of such an interaction is still lacking*.* To demonstrate the direct interactions do occur between the proximal and distal segments of ADAMTS-13, we performed a binding assay using surface plasmon resonance. As shown, following an injection of a purified recombinant ADAMTS-13 fragment (eg, MDTCS, DTCS, and MDT) over a CM5 surface coupled with a CUB fragment, the RUs (or binding) increased as a function of time during an association phase and reduced during a dissociation phase. On fitting the data of maximum RUs as a function of concentration of an ADAMTS-13 variant, we were able to obtain the binding affinity. As shown, recombinant ADAMTS-13 variants MDTCS and DTCS, but not MDT (not shown), bound to immobilized CUB domains with dissociation constants at equilibrium (*K*_*D*_) of 0.33 μM and 0.46 μM, respectively (Figure 8). These results confirmed the direct binding interactions between the distal CUB domains and proximal CysR and spacer domains in the full-length ADAMTS-13 protein in solution.

## 4 Discussion

The present study demonstrates that multiple peptide regions in the full-length recombinant ADAMTS-13 undergo a bimodal deuterium incorporation as a function of time at neutral pD. This indicates that the full-length ADAMTS-13 protein may exist in multiple intermediate confirmations and/or undergo conformational changes in solution. Absence of bimodal representation in the truncated ADAMTS-13 variants suggests that conformational transition in the variants does not occur as a function of time. This conclusion is supported by the observations with small angle X-ray scattering and cryo-EM studies [32,58,60]. In these studies, a full-length recombinant ADAMTS-13 protein appears in various shapes: curved (or “closed”) with the maximum dimensions of ∼140 Å, which may undergo transition into multiple intermediate species and more extended (or “open”) conformations, estimated to be ∼500 Å [32]. Interestingly, plasma ADAMTS-13 in healthy individuals appears to primarily exhibit the “closed” conformation, whereas it exists largely in an “open” conformation in patients with acute iTTP [61]. However, how ADAMTS-13 changes its conformation (or gets activated) under pathophysiological conditions remains poorly understood.

Previous studies have demonstrated that binding of VWF, particularly through its D4 domain [36,60] or a monoclonal antibody against the distal domains of ADAMTS-13 [32,36], to a full-length ADAMTS-13 may induce its conformational changes and increase ADAMTS-13 activity. In addition, lowering pH in the assay buffers (to 6.0) can increase ADAMTS-13 activity by ∼3-fold [32]. Low pH may disrupt the close contact between the distal and proximal domains of ADAMTS-13, thus removing the allosteric inhibition of ADAMTS-13 by the distal C-terminal domains [32]. This hypothesis is supported by the increase of proteolytic activity and the loss of pH-dependent regulation of the MDTCS, which lacks the distal inhibitory domains [32].

Previous studies also suggest that the integrin recognition sequence RGD in the CysR domain may play a role in overall activity of ADAMTS-13. These hydrophilic or charged residues are important for VWF recognition. Usually, R498 side chain is buried but shows a pH8.0 preference in our study. Part of the U-loop (ie, residue 512 and hydrophobic pockets (G471-V474) also show a pH-induced change, which may affect the overall proteolytic efficiency.

Consistent with the biochemical and functional data in the literature, our HX-MS results further reveal the bimodal deuterium incorporation behavior in various peptides of the full-length ADAMTS-13, which is absent in the truncated ADAMTS-13 variant (eg, MDTCS or T5C). More interestingly, most of these peptides derived from the CysR and spacer domains, as well as from the CUB domains in the truncated fragments, exhibit an increased initial and/or accelerated rate of deuterium incorporation as a function of time and lost pD-dependent deuterium incorporation. These results clearly indicate that in the absence of domain–domain interactions between the proximal and distal domains of ADAMTS-13, these peptides are more exposed to the surface and more accessible to the deuterium. Such a conformational change may explain why MDTCS has more proteolytic activity toward VWF substrates [62].

Moreover, overall folding of the CUB domain is highly dependent on amino acid residues such as L1221, E1267, V1270, G1302, F1304, and G1305 [59]. Similarly, the peptide regions reported in our study contains 2 (eg, E1387 and E1389) of the 6 amino acids responsible for spacer-CUB interaction [59]. In addition, our current result supports their identification of amino acid residues that had an intermediate effect (eg, K1265 and E1382) on spacer-CUB binding [59]. Overall activity of ADAMTS-13 at a given pH depends on its proton change. It is well known about the role of histidines (eg, H224, H228, and H234) and E225 for ADAMTS-13 activity. Optimum substrate binding needs rotational and translational freedom that is greatly influenced by the overall pH changes. In addition, histidine residues in the spacer domain (especially, 556-685) also play a significant role on its pH-dependent activity. The pH preference and bimodal changes in this region support the hypothesis in our study. Because we were unable to get a peptide region containing the H1364 in the CUB domain, we could not determine the overall protonation effect of this residue that may be responsible for an enhanced ADAMTS-13 activity at pH6.0 [59].

It remains to be determined where exactly the distal domains interact with the proximal domains. It is possible that the distal CUB interacts strongly with the CysR and spacer domain, but weakly with other proximal domains including the metalloprotease domain. Evidence to support this hypothesis includes an increase in deuterium accessibility in the CysR and spacer domain in the MDTCS fragment with the distal domains being removed; the CUB domains bind directly to MDTCS and DTCS with a high affinity, but only weakly to MDT; site-directed mutation in the spacer domain (R568K/F592Y/R660K/Y661F/Y665F) appears to disrupt the distal domain interaction with the proximal domains, resulting in increased proteolytic activity [36,37]; more recently, Schelpe et al. [63] demonstrate that anti-spacer (3E4) and anti-CUB domain (17G2) antibodies have the same activating activity, but not synergistically. It is believed that these antibodies may alter the catalytic efficiency of the metalloprotease domain. Consistent with this notion, a removal of the distal domains results in a reduced rate of deuterium incorporation in the metalloprotease. This may alter the substrate recognition and catalytic efficiency of the metalloprotease domain.

Comparison of truncated and full-length variants, as well as the bimodal distribution pattern, suggests the interaction of distal C-terminal domains with multiple discrete sites in the proximal domains of ADAMTS-13, resulting in protection from hydrogen–deuterium exchange. Increased deuterium uptake in the truncated variants at pH7.0 indicates quite exposed regions in them. ADAMTS-13 spacer exosite3 (eg, R659-Y665) interact with VWF-A2 domain (eg, 1651-1668), whereas the exosite in the CysR and disintegrin domains (G471-V474) and (R349 and L350) may play a central role in recognizing VWF. These residues in the full-length ADAMTS-13 are not fully accessible to deuterium exchange while truncated variants (eg, MDTCS) shows a relatively higher deuterium uptake than the full-length in various peptides.

Our HX-MS findings provide a model structure where no experimental structures are available. The results provide further structural insight into ADAMTS-13 functions, which are essential for our understandings of the protease action in normal and pathological conditions. In addition, it supports further development of rationally designed therapeutics for TTP. Our future goal is to use this technique and information to elucidate the structure of specific ADAMTS-13 epitopes prone to the development of autoimmune responses and /or mapping of antibody epitopes. Highly sensitive and accurate HX-MS technique is compliment to other structural approaches and needs very little sample volume and has no limitation in protein size or class. It can be effectively applicable to challenging protein systems such as ADAMTS-13.

Although HX-MS/MS can theoretically provide the best resolution and fastest analysis, there are a few drawbacks associated with: (1) it needs low temperature separation and high chromatography efficiency; (2) it is necessary to obtain high overlapping sequence coverage; (3) it needs a more efficient fragmentation system to obtain high resolution of peptide identification; (4) additionally, the glycosylation in the truncated vs. full-length ADAMTS-13 protein may be subtly different because they are expressed from 2 different cell lines; and (5) finally, certain areas without sufficient peptide recovery are difficult to assess for the deuterium incorporation, and pH change may result in protonation of certain residues within the domain, leading to conformational changes that are not entirely affected by the distal domain truncation.

Nevertheless, this is for the first time that we are able to provide direct evidence that conformational changes occur at various proximal regions of ADAMTS-13 following its removal of the distal domains or lowering the pH (or pD). Such a conformational change may help expose the cryptic binding sites of ADAMTS-13 for substrate recognition and may alter its catalytic site in the metalloprotease domain, thus resulting in an enhanced proteolytic activity. Our results provide novel insight into the molecular mechanism underlying how domain–domain interaction or changes in pH environment may regulate ADAMTS-13 function.

## References

[1] Zheng X., Chung D., Takayama T.K., Majerus E.M., Sadler J.E., Fujikawa K. (2001). Structure of von Willebrand factor-cleaving protease (ADAMTS13), a metalloprotease involved in thrombotic thrombocytopenic purpura. J Biol Chem.

[2] Levy G.G., Nichols W.C., Lian E.C., Foroud T., McClintick J.N., McGee B.M. (2001). Mutations in a member of the ADAMTS gene family cause thrombotic thrombocytopenic purpura. Nature.

[3] Uemura M., Tatsumi K., Matsumoto M., Fujimoto M., Matsuyama T., Ishikawa M. (2005). Localization of ADAMTS13 to the stellate cells of human liver. Blood.

[4] Cao W.J., Niiya M., Zheng X.W., Shang D.Z., Zheng X.L. (2008). Inflammatory cytokines inhibit ADAMTS13 synthesis in hepatic stellate cells and endothelial cells. J Thromb Haemost.

[5] Turner N., Nolasco L., Tao Z., Dong J.F., Moake J. (2006). Human endothelial cells synthesize and release ADAMTS-13. J Thromb Haemost.

[6] Kling S.J., Judd C.A., Warner K.B., Rodgers G.M. (2008). Regulation of ADAMTS13 expression in proliferating human umbilical vein endothelial cells. Pathophysiol Haemost Thromb.

[7] Liu L., Choi H., Bernardo A., Bergeron A.L., Nolasco L., Ruan C. (2005). Platelet-derived VWF-cleaving metalloprotease ADAMTS-13. J Thromb Haemost.

[8] Dong J.F., Moake J.L., Nolasco L., Bernardo A., Arceneaux W., Shrimpton C.N. (2002). ADAMTS-13 rapidly cleaves newly secreted ultralarge von Willebrand factor multimers on the endothelial surface under flowing conditions. Blood.

[9] López J.A., Dong J.F. (2004). Cleavage of von Willebrand factor by ADAMTS-13 on endothelial cells. Semin Hematol.

[10] Tsai H.M. (1996). Physiologic cleavage of von Willebrand factor by a plasma protease is dependent on its conformation and requires calcium ion. Blood.

[11] Zheng X.L. (2015). ADAMTS13 and von Willebrand factor in thrombotic thrombocytopenic purpura. Annu Rev Med.

[12] Furlan M., Robles R., Galbusera M., Remuzzi G., Kyrle P.A., Brenner B. (1998). Von Willebrand factor-cleaving protease in thrombotic thrombocytopenic purpura and the hemolytic-uremic syndrome. N Engl J Med.

[13] Kaikita K., Soejima K., Matsukawa M., Nakagaki T., Ogawa H. (2006). Reduced von Willebrand factor-cleaving protease (ADAMTS13) activity in acute myocardial infarction. J Thromb Haemost.

[14] Matsukawa M., Kaikita K., Soejima K., Fuchigami S., Nakamura Y., Honda T. (2007). Serial changes in von Willebrand factor-cleaving protease (ADAMTS13) and prognosis after acute myocardial infarction. Am J Cardiol.

[15] Bongers T.N., de Maat M.P., van Goor M.L., Bhagwanbali V., van Vliet H.H.D.M., Gómez García E.B. (2006). High von Willebrand factor levels increase the risk of first ischemic stroke: influence of ADAMTS13, inflammation, and genetic variability. Stroke.

[16] Andersson H.M., Siegerink B., Luken B.M., Crawley J.T.B., Algra A., Lane D.A. (2012). High VWF, low ADAMTS13, and oral contraceptives increase the risk of ischemic stroke and myocardial infarction in young women. Blood.

[17] de Mast Q., Groot E., Asih P.B., Syafruddin D., Oosting M., Sebastian S. (2009). ADAMTS13 deficiency with elevated levels of ultra-large and active von Willebrand factor in P. falciparum and P. vivax malaria. Am J Trop Med Hyg.

[18] Larkin D., de Laat B., Jenkins P.V., Bunn J., Craig A.G., Terraube V. (2009). Severe Plasmodium falciparum malaria is associated with circulating ultra-large von Willebrand multimers and ADAMTS13 inhibition. PLOS Pathog.

[19] Löwenberg E.C., Charunwatthana P., Cohen S., van den Born B.J., Meijers J.C., Yunus E.B. (2010). Severe malaria is associated with a deficiency of von Willebrand factor cleaving protease, ADAMTS13. Thromb Haemost.

[20] Stepanian A., Cohen-Moatti M., Sanglier T., Legendre P., Ameziane N., Tsatsaris V. (2011). Von Willebrand factor and ADAMTS13: a candidate couple for preeclampsia pathophysiology. Arterioscler Thromb Vasc Biol.

[21] Aref S., Goda H. (2013). Increased VWF antigen levels and decreased ADAMTS13 activity in preeclampsia. Hematology.

[22] Ono T., Mimuro J., Madoiwa S., Soejima K., Kashiwakura Y., Ishiwata A. (2006). Severe secondary deficiency of von Willebrand factor-cleaving protease (ADAMTS13) in patients with sepsis-induced disseminated intravascular coagulation: its correlation with development of renal failure. Blood.

[23] Rodríguez M., Castro Quismondo N., Zafra Torres D., Gil Alos D., Ayala R., Martinez-Lopez J. (2021). Increased von Willebrand factor antigen and low ADAMTS13 activity are related to poor prognosis in Covid-19 patients. Int J Lab Hematol.

[24] Delrue M., Siguret V., Neuwirth M., Joly B., Beranger N., Sène D. (2021). Von Willebrand factor/ADAMTS13 axis and venous thromboembolism in moderate-to-severe COVID-19 patients. Br J Haematol.

[25] Martinelli N., Montagnana M., Pizzolo F., Friso S., Salvagno G.L., Forni G.L. (2020). A relative ADAMTS13 deficiency supports the presence of a secondary microangiopathy in COVID 19. Thromb Res.

[26] Tsai H.M., Sussman, Nagel R.L. (1994). Shear stress enhances the proteolysis of von Willebrand factor in normal plasma. Blood.

[27] Cao W., Krishnaswamy S., Camire R.M., Lenting P.J., Zheng X.L. (2008). Factor VIII accelerates proteolytic cleavage of von Willebrand factor by ADAMTS13. Proc Natl Acad Sci U S A.

[28] Skipwith C.G., Cao W., Zheng X.L. (2010). Factor VIII and platelets synergistically accelerate cleavage of von Willebrand factor by ADAMTS13 under fluid shear stress. J Biol Chem.

[29] Nishio K., Anderson P.J., Zheng X.L., Sadler J.E. (2004). Binding of platelet glycoprotein Ibalpha to von Willebrand factor domain A1 stimulates the cleavage of the adjacent domain A2 by ADAMTS13. Proc Natl Acad Sci U S A.

[30] Cao W., Abdelgawwad M.S., Li J., Zheng X.L. (2019). Apolipoprotein B100/low-density lipoprotein regulates proteolysis and functions of von Willebrand factor under arterial shear. Thromb Haemost.

[31] Zhu J., Muia J., Gupta G., Westfield L.A., Vanhoorelbeke K., Tolia N.H. (2019). Exploring the “minimal” structure of a functional ADAMTS13 by mutagenesis and small-angle X-ray scattering. Blood.

[32] Muia J., Zhu J., Gupta G., Haberichter S.L., Friedman K.D., Feys H.B. (2014). Allosteric activation of ADAMTS13 by von Willebrand factor. Proc Natl Acad Sci USA.

[33] Anderson P.J., Kokame K., Sadler J.E. (2006). Zinc and calcium ions cooperatively modulate ADAMTS13 activity. J Biol Chem.

[34] Han Y., Xiao J., Falls E., Zheng X.L. (2011). A shear-based assay for assessing plasma ADAMTS13 activity and inhibitors in patients with thrombotic thrombocytopenic purpura. Transfusion.

[35] South K., Freitas M.O., Lane D.A. (2017). A model for the conformational activation of the structurally quiescent metalloprotease ADAMTS13 by von Willebrand factor. J Biol Chem.

[36] South K., Luken B.M., Crawley J.T., Phillips R., Thomas M., Collins R.F. (2014). Conformational activation of ADAMTS13. Proc Natl Acad Sci USA.

[37] Jian C., Xiao J., Gong L., Skipwith C.G., Jin S.Y., Kwaan H.C. (2012). Gain-of-function ADAMTS13 variants that are resistant to autoantibodies against ADAMTS13 in patients with acquired thrombotic thrombocytopenic purpura. Blood.

[38] Bruno K., Völkel D., Plaimauer B., Antoine G., Pable S., Motto D.G. (2005). Cloning, expression and functional characterization of the full-length murine ADAMTS13. J Thromb Haemost.

[39] Cao W.J., Zheng X.L. (2017). Conformational quiescence of ADAMTS-13 prevents proteolytic promiscuity: comment. J Thromb Haemost.

[40] Xiao J., Jin S.Y., Xue J., Sorvillo N., Voorberg J., Zheng X.L. (2011). Essential domains of a disintegrin and metalloprotease with thrombospondin type 1 repeats-13 metalloprotease required for modulation of arterial thrombosis. Arterioscler Thromb Vasc Biol.

[41] Ai J., Smith P., Wang S., Zhang P., Zheng X.L. (2005). The proximal carboxyl-terminal domains of ADAMTS13 determine substrate specificity and are all required for cleavage of von Willebrand factor. J Biol Chem.

[42] Englander J.J., Del Mar C., Li W., Englander S.W., Kim J.S., Stranz D.D. (2003). Protein structure change studied by hydrogen-deuterium exchange, functional labeling, and mass spectrometry. Proc Natl Acad Sci U S A.

[43] Krishna M.M., Hoang L., Lin Y., Englander S.W. (2004). Hydrogen exchange methods to study protein folding. Methods.

[44] Casina V.C., Hu W., Mao J.H., Lu R.N., Hanby H.A., Pickens B. (2015). High-resolution epitope mapping by HX MS reveals the pathogenic mechanism and a possible therapy for autoimmune TTP syndrome. Proc Natl Acad Sci USA.

[45] Hu W., Walters B.T., Kan Z.Y., Mayne L., Rosen L.E., Marqusee S. (2013). Stepwise protein folding at near amino acid resolution by hydrogen exchange and mass spectrometry. Proc Natl Acad Sci U S A.

[46] Kan Z.Y., Mayne L., Chetty P.S., Englander S.W. (2011). ExMS: data analysis for HX-MS experiments. J Am Soc Mass Spectrom.

[47] Zhang P., Pan W., Rux A.H., Sachais B.S., Zheng X.L. (2007). The cooperative activity between the carboxyl-terminal TSP1 repeats and the CUB domains of ADAMTS13 is crucial for recognition of von Willebrand factor under flow. Blood.

[48] Plaimauer B., Zimmermann K., Völkel D., Antoine G., Kerschbaumer R., Jenab P. (2002). Cloning, expression, and functional characterization of the von Willebrand factor-cleaving protease (ADAMTS13). Blood.

[49] Plaimauer B., Kremer Hovinga J.A., Juno C., Wolfsegger M.J., Skalicky S., Schmidt M. (2011). Recombinant ADAMTS13 normalizes von Willebrand factor-cleaving activity in plasma of acquired TTP patients by overriding inhibitory antibodies. J Thromb Haemost.

[50] Viel K.R., Machiah D.K., Warren D.M., Khachidze M., Buil A., Fernstrom K. (2007). A sequence variation scan of the coagulation factor VIII (FVIII) structural gene and associations with plasma FVIII activity levels. Blood.

[51] Kan Z.Y., Walters B.T., Mayne L., Englander S.W. (2013). Protein hydrogen exchange at residue resolution by proteolytic fragmentation mass spectrometry analysis. Proc Natl Acad Sci USA.

[52] Zheng X., Nishio K., Majerus E.M., Sadler J.E. (2003). Cleavage of von Willebrand factor requires the spacer domain of the metalloprotease ADAMTS13. J Biol Chem.

[53] Gao W., Anderson P.J., Majerus E.M., Tuley E.A., Sadler J.E. (2006). Exosite interactions contribute to tension-induced cleavage of von Willebrand factor by the antithrombotic ADAMTS13 metalloprotease. Proc Natl Acad Sci USA.

[54] Gao W., Zhu J., Westfield L.A., Tuley E.A., Anderson P.J., Sadler J.E. (2012). Rearranging exosites in noncatalytic domains can redirect the substrate specificity of ADAMTS proteases. J Biol Chem.

[55] Luken B.M., Turenhout E.A., Hulstein J.J., Van Mourik J.A., Fijnheer R., Voorberg J. (2005). The spacer domain of ADAMTS13 contains a major binding site for antibodies in patients with thrombotic thrombocytopenic purpura. Thromb Haemost.

[56] Pos W., Luken B.M., Kremer Hovinga J.A., Turenhout E.A., Scheiflinger F., Dong J.F. (2009). VH1-69 germline encoded antibodies directed towards ADAMTS13 in patients with acquired thrombotic thrombocytopenic purpura. J Thromb Haemost.

[57] Pos W., Sorvillo N., Fijnheer R., Feys H.B., Kaijen P.H.P., Vidarsson G. (2011). Residues Arg568 and Phe592 contribute to an antigenic surface for anti-ADAMTS13 antibodies in the spacer domain. Haematologica.

[58] Muia J., Zhu J., Greco S.C., Vanhoorelbeke K., Gupta G., Westfield L.A. (2019). Phylogenetic and functional analysis of ADAMTS13 identifies highly conserved domains essential for allosteric regulation. Blood.

[59] Kim H.J., Xu Y., Petri A., Vanhoorelbeke K., Crawley J.T.B., Emsley J. (2021). Crystal structure of ADAMTS13 CUB domains reveals their role in global latency. Sci Adv.

[60] South K., Freitas M.O., Lane D.A. (2018). A model for the conformational activation of the structurally quiescent metalloprotease ADAMTS13 by von Willebrand factor. J Biol Chem.

[61] Roose E., Schelpe A.S., Joly B.S., Peetermans M., Verhamme P., Voorberg J. (2018). An open conformation of ADAMTS-13 is a hallmark of acute acquired thrombotic thrombocytopenic purpura. J Thromb Haemost.

[62] Gao W., Anderson P.J., Sadler J.E. (2008). Extensive contacts between ADAMTS13 exosites and von Willebrand factor domain A2 contribute to substrate specificity. Blood.

[63] Schelpe A.S., Petri A., Roose E., Pareyn I., Deckmyn H., De Meyer S.F. (2020). Antibodies that conformationally activate ADAMTS13 allosterically enhance metalloprotease domain function. Blood Adv.

